# Evaluation of the level of information of pediatricians about the diagnosis and management of cryptorchidism

**DOI:** 10.1016/j.jped.2024.06.002

**Published:** 2024-07-09

**Authors:** Larissa de Lima Monte, Rodrigo Campos Ocáriz, Joaquim Murray Bustorff-Silva, Patricia Traballi de Carvalho Pegolo, Gil Guerra-Junior, Márcio Lopes Miranda

**Affiliations:** a Universidade Estadual de Campinas, Faculdade de Medicina, Departamento de Cirurgia, Divisão de Cirurgia Pediátrica, Campinas, SP, Brazil; b Universidade Estadual de Campinas, Faculdade de Medicina, Departamento de Pediatria, Divisão de Endocrinologia Pediátrica, Campinas, SP, Brazil

**Keywords:** Cryptorchidism, Diagnosis, Clinical decision-making, Surgical procedure, Orchiopexy, Pediatrics

## Abstract

**Objective:**

Evaluate the level of information of pediatricians about the diagnosis and management of cryptorchidism.

**Method:**

A cross-sectional observational study was conducted using a form via the "Google Forms" platform. The study population included pediatricians and pediatric residents associated with the Brazilian Society of Pediatrics. Seven hundred twenty-eight responses were recorded and analyzed using IBM SPSS v21.

**Results:**

728 valid responses were obtained. Of these answers, only 20.5 % answered that the physical examination was sufficient for the diagnosis, and 79.4 % responded that they requested ultrasound as the best test to aid in diagnosing cryptorchidism. When questioned about the ideal age for referring a patient with cryptorchidism, the survey recorded 56.3 % of the responses defending the correct age as six months old, 30.2 % shortly after birth, and 13.2 % at two years old. Other topics were addressed in the form, such as the frequency of evaluation of testicular position and investigation for DDS, among others. Still, the answers to these questions were compatible with current manuals and guidelines on cryptorchidism.

**Conclusion:**

It is evident that the understanding of the professionals consulted about the diagnosis and management of cryptorchidism needs to be updated with the current practices adopted and that pediatricians, in general, must maintain periodic programs on this subject. Therefore, this topic should be part of a continuing education program with pediatric surgery.

## Introduction

Cryptorchidism is the most common genitourinary anomaly in male infants, and it is defined as a testicle located outside the scrotum and at any point in its normal migration path.^1^ The incidence is variable and depends on factors such as gestational age, affecting 1.0–4.6 % of term infants and 1.1–45 % of preterm neonates.^2^ According to the Information System on Live Births (SINASC), in Brazil in 2020, 444 undescended testes were registered, corresponding to 1.88 % of the congenital anomalies reported in the same year.^3^ Apparently, the prevalence of this disease is increasing, but this data is possibly related to the increased survival of extremely premature and small-for-gestational-age babies.^4^ Cryptorchidism may be associated with disorders of sexual development and congenital malformation, but it is mainly found as an isolated malformation in up to 85 % of cases.^5^

It is known that testes descent is related to factors such as testicular enlargement, increased intra-abdominal pressure, hormonal action, and growth of the cranial part of the abdomen moving away from the future pelvic region.^6^ When this migration does not occur during pregnancy, it can still happen in the first six months of life due to hormonal activity. Hence, intervention is not recommended before this age.^7^

Regarding the complications associated with cryptorchidism, a reduction of germ cells has been observed in patients with cryptorchidism after one year of age. Also, there is a greater risk of developing germ cell tumors in adolescent patients. It is known that men with a history of this disorder have an increased risk of cancer. Studies point to an increased incidence of malignancy in cryptorchid testes ranging from 49/100,000 (0.05 %) to 12/1075 (1 %).^8^^,^^9^

The diagnosis is clinical, and a thorough pediatric genital physical examination is sufficient to detect cryptorchidism. Ultrasonography is not recommended, as this method does not reliably differentiate cryptorchidism from retractile testicles, wasting resources and potentially delaying surgical correction.^4^^,^^10^

**Figure 1:**
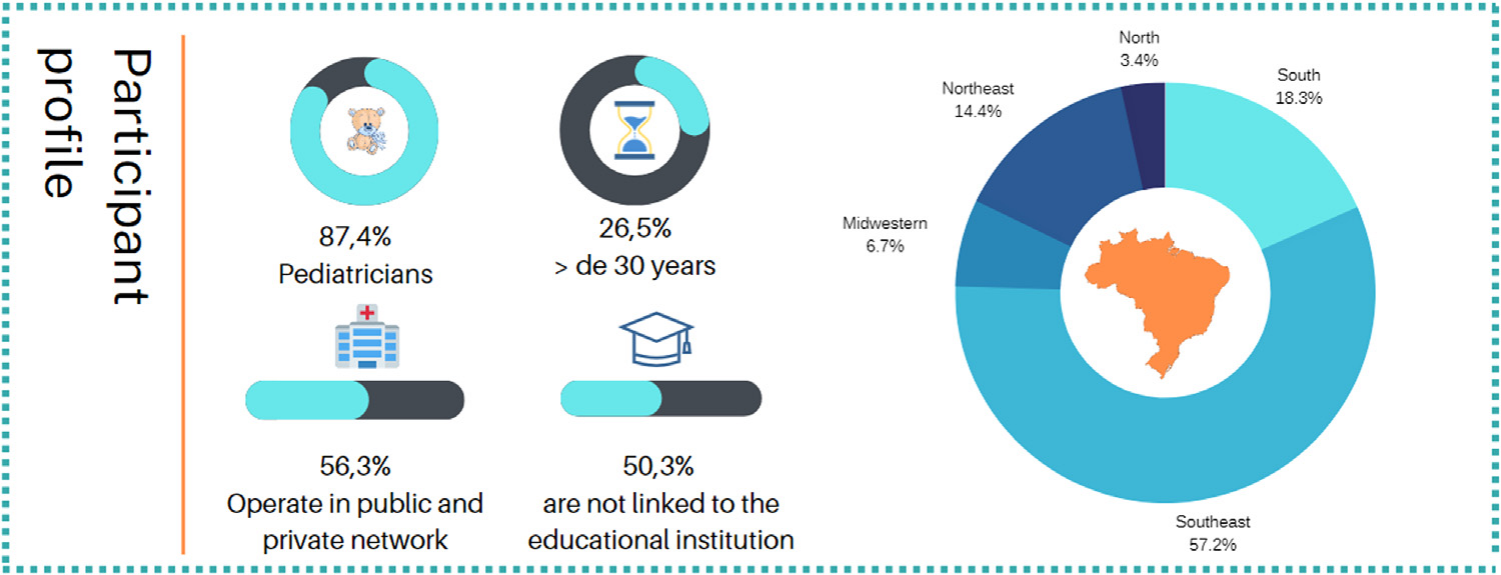
Summary graphs of the survey participants' profiles.

Surgery is considered more effective than hormones and is recommended for babies whose testicles did not descend until six months of age.^2^^,^^11^ Depending on the location of the testicle, a specific surgical approach is indicated. In cases of abdominal testes, laparoscopy helps in diagnosis and therapy^12^^,^^13^ (Figure 1). However, it is not certain if the information regarding the best age to operate has reached the pediatricians, who are the first to diagnose an undescended testis and refer the patient to the surgeons.

### Aim

This study aims to investigate the level of information pediatricians have about the subject

## Population and methods

A cross-sectional observational study was designed to investigate the management of undescended testes by health professionals attending to children. A set of questions was prepared on the diagnosis and management of cryptorchidism. Therefore, the final form was applied via "Google Forms," containing 15 questions, with only one correct alternative. The protocol was submitted and approved by the Local Ethics Committee (CAAE 47,886,321.6.0000.5404).

This form was sent to pediatricians and pediatric residents, members of the Brazilian Society of Pediatrics (SBP). The invitation letter with the link to the form was sent to the participants via email by the SBP. According to the SBP mailing report, 18,577 emails were sent, of which only 29,1 % were opened.

A total of 762 participants answered the form, with 16 duplicated responses, 13 non-pediatrician participants, and 5 participants did not accept the Informed Consent Form, so these participants did not respond to the form, totaling 728 answers.

Initially, the responses were stored in a Microsoft Excel spreadsheet, and the graphics provided by the Google Form platform were recorded. A statistical study used the IBM SPSS version 22 computer program to describe the variables.

## Results

The present study revealed that, regarding the profile of the participants, there was a predominance of participants who declared themselves to be pediatricians (87.4 %), with 10.2 % residents in pediatrics and 2.5 % residents in pediatric specialties. Regarding the years of training of the participants, there was a slight predominance of those with more than 30 years of training (26.5 %). Approximately half of the interviewees are not linked to a pediatrics teaching institution. Among those who declared having a link with an educational institution, 23.1 % are medical assistants, 16.6 % work as professors, and 10.6 % are residents of these institutions. Most of the participants came from the southeast of the country. Most participants stated that they work in both public and private networks.

In the block of general questions on the topic, the frequencies described below in Table 1 were recorded, and it was possible to observe that most participants selected the alternative that corresponded to the most consensual answers between societies.

**Table 1:** Answers obtained in the study. Refer to questions and alternatives in full in the appendix.

**Questions included in the study**					
Question	Answer (percentage)				
	A	B	C	D	E
Periodicity of testicular exam	11,5	87,9*	0,5	–	–
Complementary exam	79	4	1	20,5*	–
Surgery referral age	30,2	56,3*	13,2	0,3	–
The ideal age for surgery	10,2	47,4*	33,7	8,8	–
Use of hormone therapy	92,4*	3,4	1,6	0,7	1,8
Retractile testicle treatment	29,4*	23,8	17,6	1,2	28
Difficulty in referral	64,8	12,2	22,1	0,8	–
The main objective of orchidopexy	0,3	87,9*	3,8	8	–

⁎ Indicates the alternative that corresponded to the most consensual answers by the clinical societies.

However, two main survey points were highlighted in the final analysis of the data. The first concerns the frequency of professionals requesting complementary exams to diagnose cryptorchidism, with 79 % of participants indicating using ultrasound to confirm the diagnosis.

Another point that drew our attention was the ideal age for referral. The survey results indicated that only a little more than half of the professionals consulted are aware of the ideal age for referral (Table 2).

**Table 2:** Table of the answers to the questions "Which complementary exam do you use to help the diagnosis of cryptorchidism?", "What is the ideal age for referring a patient with cryptorchidism to the Surgeon?", and "What is the ideal age for surgery?".

What complementary test do you use to help diagnose cryptorchidism?	
Category	%
Ultrasound	79
Magnetic Resonance Imaging	0,4
Computed tomography	0,1
None, as physical examination is sufficient for diagnosis	20,5

What is the ideal age to refer a patient with cryptorchidism to the Surgeon?	
After birth	30,2
At six months of age	56,3
At two years of age	13,2
At five years of age	0,3

What is the ideal age for surgery?	
Before six months of age	10,2
Between 6 and 12 months of age	47,4
Up to 2 years of age	33,7
Up to 5 years of age	8,8

When analyzing the answers to the question about the ideal age for referral, it can be seen that responders indicating six months as the ideal age for surgery were predominantly those professionals having less than five and more than 30 years of practice and those linked to an educational institution.

Nevertheless, when the authors analyzed the data regarding the ideal age pediatricians consider suitable for operating, we observed that more than half of them chose alternatives with a different age range from 6 months to 12 months of life, as seen in Table 2.

## Discussion

Data from the present survey indicates that nearly 40 % of pediatricians still believe that the ideal age for treating cryptorchidism may exceed 12 months of age and also that almost 80 % still rely on the use of ultrasound to confirm the diagnosis. Diagnosis of cryptorchidism is clinical and depends on adequate access to health services and the technical capacity of the examiner.^10^ However, 79.4 % of the research participants responded that they use ultrasound as diagnostic support. Only 20.1 % stated that there is no need for complementary exams because the physical exam is enough for the diagnosis. The use of complementary exams, such as ultrasonography (US), is not recommended because this method does not reliably differentiate cryptorchidism from other diagnoses and does not influence the conduct, surgical approach, or evaluation of the viability of the testes involved, and neither does it rule out an intra-abdominal testicle, being a waste of resources that may lead to a delay in surgical correction.^14^ A retrospective study from Ottawa, Canada, concluded that the referral of patients with suspected undescended testis should not be accompanied by ultrasound, as it is unnecessary and misleading, in addition to consuming health resources.^15^ A prospective study by the University of Toronto revealed that ultrasound performed poorly as a diagnostic tool in detecting palpable undescended testes in boys, with a specificity of only 16 %.^16^ In this setting, radiological tests have a specificity of 44 %, usually lower than physical examination, which reaches 84 % specificity when performed by a pediatric urologist. Although magnetic resonance has greater sensitivity and specificity, it is an expensive test that is not widely available and requires sedation in pediatric patients.^17^^,^^18^

Pediatricians' performance is essential for timely diagnosis and referral to surgery.^9^ Due to the adverse clinical outcomes, it is crucial that the diagnosis be made as early as possible and that, ideally, it takes place in the delivery room.^19^ Most of the participants consulted in the survey also considered that the pediatrician should examine the newborn's testicles for the first time in the delivery room itself, accounting for 93.5 % of responses in this item. In Brazil, Ordinance Number 31 of February 15, 1993, of the Ministry of Health directs a pediatrician or neonatologist's assessment of the newborn in the delivery room until the newborn is transferred to the care of the multidisciplinary team or rooming-in. Therefore, it is up to these professionals to complete a physical examination of the newborn. Pediatricians need adequate training to identify cryptorchidism and other congenital anomalies and offer appropriate treatment earlier. In addition, the position of non-palpable testicles at birth should be reassessed in the eighth week of life and at three months of life.^20^

Misdiagnosis and late referral seem to be a widespread problem. A University of Texas study concluded that most 121 patients referred to a pediatric urologist for cryptorchidism were referred after 12 months of life, and only half of the patients presented cryptorchidism.^21^

Orchiopexy is recommended between 6 and 12 months, or a maximum of 18 months, by most societies.^13^^,^^22^ In the second edition of the Brazilian Treaty of Pediatrics, published in 2010, there was already a recommendation for orchidopexy at 12 months of life.^23^ This ideal age range was determined from the histological analysis of testicular tissue and the effects on fertility according to the time the correction was performed. Also, there is evidence of better results of the average tubular fertility index and the germ cell count in patients operated on before the first year of life.^22^^,^^24^ The present survey shows that only 47.6 % of professionals indicated six to twelve months of life as the ideal age for surgery. This data underscores the lack of up-to-date information in almost half of the consulted pediatricians. Not surprisingly, younger pediatricians (graduates of less than ten years) responded with more correct answers.

Regarding the main objective for performing orchidopexy, 88 % of professionals indicate the procedure to reduce the incidence of testicular tumors and ensure the maintenance of sperm production. Although all options bring proven benefits from this surgery, the main objective of the procedure is to provide global testicular function, in addition to other benefits, such as the prevention of trauma.^22^ Professionals must consider these benefits to prioritize early diagnosis and provide the patient and his family with relevant information.

The surgical approach is considered more effective than the use of hormones since the therapies that use hormone treatment are based on low-grade scientific evidence studies that do not assess the heterogeneity of patients, the location of the testicle, the hormone dose, and the lack of long-term studies.^22^ In addition, using hormones has short-term side effects such as scrotal erythema, pigmentation, induction of pubic hair, and penile growth, although these tend to regress with interruption of treatment. Therefore, although it has been used in special situations, like, for instance, bilateral chriptorchism, hormone therapy is currently not recommended.^2^^,^^4^^,^^22^ In the present survey, 92.3 % of professionals did not recommend hormone therapy.

This study has some limitations. The results herein expressed should be regarded with caution because the number of respondents (although a large number) represents less than 10 % of the total number of pediatricians in Brazil. Also, pediatricians who are interested in the subject might be over-represented in the population study resulting in a selection bias. Despite this, due to the overall distribution of the responders, it is believed that the results reflect roughly the present state of knowledge among these professionals about cryptorchidism. Another important limitation is that the questions and the resulting answers are not applicable to acquired cryptorchism which is a different (although not less important) clinical entity, that should also be recognized by every pediatrician.

The results of this survey indicate that pediatricians' knowledge of the diagnosis and management of cryptorchidism is outdated and does not include the more current practices. These results show the importance of maintaining periodic update programs for pediatricians in general, involving educational institutions, medical societies, and health professionals.

## Conflicts of interest

The authors declare no conflicts of interest.

## Appendix - Formulary and percentage of response obtained for each question

1. What is your level of vocational education?
  A. Pediatrics resident (10,2 %)
  B. Pediatrics subspecialty resident (2,5 %)
  C. Pediatrician (87,4 %)
2. How many years since graduation?
  A. < 5 years (17,4 %)
  B. 5 to 10 years (15,5 %)
  C. 10 to 20 years^20^^,^^5^
  D. 20 to 30 years (20,1 %)
  E. > 30 years (26,5 %)
3. Are you linked to a teaching institution in Pediatrics?
  A. Yes, as a resident (10,6 %)
  B. Yes, as a teacher (16,6 %)
  C. Yes, as a physician assistant (23,1 %)
  D. No (49,7 %)
4. In which region did Mr. (Mrs.) act?
  A. North (3,4 %)
  B. North East (14,4 %)
  C. Midwest (6,7 %)
  D. Southeast (57,7 %)
  E. South (18,3 %)
5. Do you work in the public or private network?
  A. Public and private network (56,3 %)
  B. Public network only (19,5 %)
  C. Private network only (24,2 %)
6. What name do you use for changes in testicular descent?
  A. Cryptorchidism (69,9 %)
  B. Testicular dystopia (21,4 %)
  C. Undescended testicle (7 %)
  D. Empty scrotum syndrome (1,6 %)
7. Do you examine the testicles at every doctor's appointment?
  A. Yes, only on the first consultation (11,5 %)
  B. Yes, annually (87,9 %)
  C. No, as the examination of the testicles is the specialist's responsibility (0,5 %).
8. What complementary exam do you use to help diagnose cryptorchidism?
  A. Ultrasound (79,0 %)
  B. Magnetic resonance (0,4 %)
  C. Computed tomography (0,1 %)
  D. None, as physical examination is sufficient for diagnosis (20,5 %)
9. What is the ideal age for referring a patient with cryptorchidism to a surgeon?
  A. After birth (30,2 %)
  B. At six months of age (56,3 %)
  C. At two years of age (13,2 %)
  D. After five years of life (0,3 %)
10. What is the ideal age for surgery?
  A. Before six months of age (10,2 %)
  B. Between 6 and 12 months of age (47,4 %)
  C. Up to 2 years of age (33,7 %)
  D. Up to 5 years of age (8,8 %)
11. Do you use hormone therapy to stimulate testicular descent?
  A. Never (92,4 %)
  B. Yes, only in bilateral cryptorchid testicles (3,4 %)
  C. Yes, as a surgery facilitator (1,6 %)
  D. Yes, only in abdominal testicles (0,7 %)
  E. Yes, only for retractile testicle (1,8 %)
12. Do you consider retractile testicles a disease that must be treated?
  A. No (29,4 %)
  B. Yes, in all cases (23,8 %)
  C. Yes, after five years of age (17,6 %)
  D. Yes, when small testicles (1,2 %)
  E. Yes, in specific cases (e.g., chronic pain attributed to the retractile testicle) (28,0 %)
13. What is the best method to find a non-palpable testicle?
  A. Ultrasound (67,9 %)
  B. Computed tomography (6,0 %)
  C. Magnetic resonance (10,3 %)
  D. Videolaparoscopy (15,8 %)
14. Do you encounter any difficulties referring your patients with cryptorchidism?
  A. No (64,8 %)
  B. Yes, due to the lack of specialized professionals (12,2 %)
  C. Yes, due to the ineffective public system (22,1 %)
  D. Yes, due to little cooperation from families (0,8 %)
15. What is the main objective of performing orchidopexy?
  A. Aesthetic (0,3 %)
  B. Reduce the incidence of testicular tumors and maintain sperm production (87,9 %)
  C. Maintain hormone production (3,8 %)
  D. Prevent testicular torsion (8,0 %)
